# Single-Molecule Fluorescence Reveals the Unwinding Stepping Mechanism of Replicative Helicase

**DOI:** 10.1016/j.celrep.2014.02.022

**Published:** 2014-03-13

**Authors:** Salman Syed, Manjula Pandey, Smita S. Patel, Taekjip Ha

**Affiliations:** 1 Center for Biophysics and Computational Biology, University of Illinois at Urbana-Champaign, Urbana, IL 61801, USA; 2 Department of Biochemistry and Molecular Biology, Rutgers-Robert Wood Johnson Medical School, Piscataway, NJ 08854, USA; 3 Department of Physics and Center for the Physics of Living Cells, University of Illinois at Urbana-Champaign, Urbana, IL 61801, USA; 4 Howard Hughes Medical Institute, Urbana, IL 61801, USA

## Abstract

Bacteriophage T7 gp4 serves as a model protein for replicative helicases that couples deoxythymidine triphosphate (dTTP) hydrolysis to directional movement and DNA strand separation. We employed single-molecule fluorescence resonance energy transfer methods to resolve steps during DNA unwinding by T7 helicase. We confirm that the unwinding rate of T7 helicase decreases with increasing base pair stability. For duplexes containing >35% guanine-cytosine (GC) base pairs, we observed stochastic pauses every 2–3 bp during unwinding. The dwells on each pause were distributed nonexponentially, consistent with two or three rounds of dTTP hydrolysis before each unwinding step. Moreover, we observed backward movements of the enzyme on GC-rich DNAs at low dTTP concentrations. Our data suggest a coupling ratio of 1:1 between base pairs unwound and dTTP hydrolysis, and they further support the concept that nucleic acid motors can have a hierarchy of different-sized steps or can accumulate elastic energy before transitioning to a subsequent phase.

## Highlights

• Single DNA unwinding assay recapitulates sequence-dependent unwinding
• High-resolution data reveal an unwinding step size of 2–3 bp
• Two or three hidden steps precede the unwinding step, suggesting 1:1 chemical coupling
• 1:1 coupling is maintained at low dNTP, but helicase often slips backward

## Graphical Abstract

**Figure:**
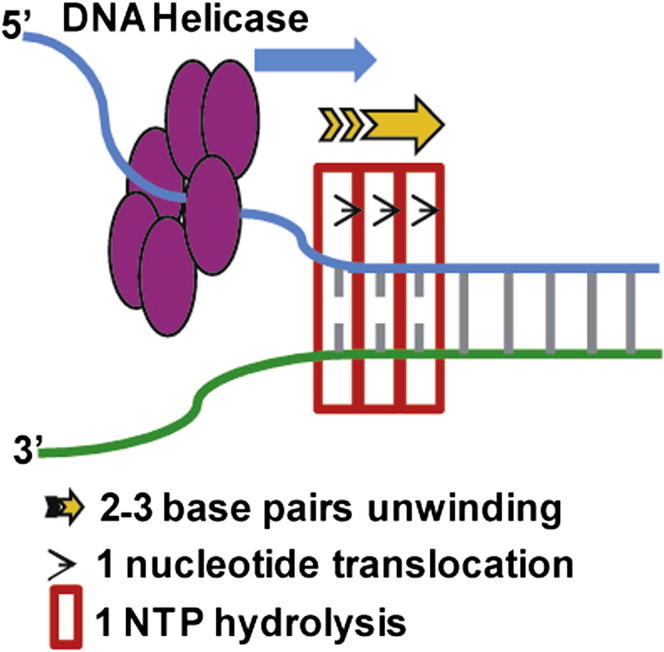

## Teaser

Ha and colleagues provide direct evidence that the T7 helicase unwinds DNA in discrete steps of 2–3 bp. Each unwinding step consists of two or three hidden steps, suggesting a coupling ratio of 1:1 between base pairs unwound and dNTP hydrolyzed, and indicating accumulation of elastic energy, before transitioning to a subsequent phase. At low dNTP, the helicase also displays backward movements, likely due to loss of subunit coordination.

## Introduction

DNA helicases are motor enzymes that convert the chemical energy of nucleotide triphosphate hydrolysis into mechanical energy for translocation on single-stranded DNA (ssDNA) and unwinding of double-stranded DNA (dsDNA) (Lohman and Bjornson, 1996, Patel and Picha, 2000). These enzymes encounter DNA sequences of different stabilities, and studying the effect of base pair stability can provide insights into the unwinding mechanisms (Betterton and Jülicher, 2005, von Hippel and Delagoutte, 2001). Previous studies have shown that the unwinding rates of T7 helicase, T4 helicase, and hepatitis virus C NS3 helicase depend on the stability of nucleic acid base pairs (Donmez et al., 2007, Johnson et al., 2007, Lionnet et al., 2007, Cheng et al., 2007).

T7 helicase (gp4A′ protein) has served as a good model system for hexameric helicases (Patel and Hingorani, 1993, Richardson, 1983). It assembles into a ring-shaped hexamer in the presence of deoxythymidine triphosphate (dTTP) and ssDNA (Donmez and Patel, 2006, Egelman et al., 1995), and translocates on ssDNA in the 5′ to 3′ direction. It unwinds dsDNA using a strand-exclusion mechanism whereby it binds and moves along one strand of the dsDNA in the 5′ to 3′ direction while excluding the complementary strand from its central channel (Ahnert and Patel, 1997, Hacker and Johnson, 1997, Jezewska et al., 1998, Kaplan and O’Donnell, 2002).

Using ensemble single-turnover kinetic analysis, Jeong et al. (2004) estimated the kinetic step size of unwinding by T7 helicase to be ∼10 bp. As defined, the kinetic step size provides an estimate of how often a recurrent rate-limiting step takes place during processive unwinding. However, the kinetic step size estimated from ensemble measurements can be inflated if there exists significant heterogeneity in the reaction rate among individual molecules (Park et al., 2010). In addition, the crystal structures of hexameric helicases published thus far have not given detailed information on the unwinding mechanisms because either the nucleic acid substrates were not included in the structures (Bailey et al., 2007, Gai et al., 2004, Li et al., 2003, Singleton et al., 2000, Wang et al., 2008) or only the single-stranded substrates were cocrystallized (Enemark and Joshua-Tor, 2006, Itsathitphaisarn et al., 2012, Thomsen and Berger, 2009). Although single-molecule techniques have provided detailed insights into the mechanisms of various helicases (Bianco et al., 2001, Cheng et al., 2007, Cheng et al., 2011, Dessinges et al., 2004, Dohoney and Gelles, 2001, Fili et al., 2010, Ha et al., 2002, Honda et al., 2009, Johnson et al., 2007, Karunatilaka et al., 2010, Klaue et al., 2013, Lee et al., 2006, Lionnet et al., 2007, Manosas et al., 2009, Myong et al., 2005, Myong et al., 2007, Myong et al., 2009, Park et al., 2010, Perkins et al., 2004, Qi et al., 2013, Spies et al., 2003, Sun et al., 2011), unwinding steps have not been detected for any hexameric helicase. In this report, we used single-molecule fluorescence resonance energy transfer (smFRET) (Ha et al., 1996) to measure real-time DNA unwinding by individual T7 helicase molecules. Taking advantage of the sequence-dependent unwinding rate, we used a designed DNA substrate to find a relation between FRET efficiency and the number of base pairs unwound. From substrates with a high guanine-cytosine (GC) content, we could detect the individual steps of DNA unwinding and analyze their kinetics.

## Results

### smFRET Unwinding Assay Shows the Duplex Stability Dependence of the Unwinding Rate

**Figure 1:**
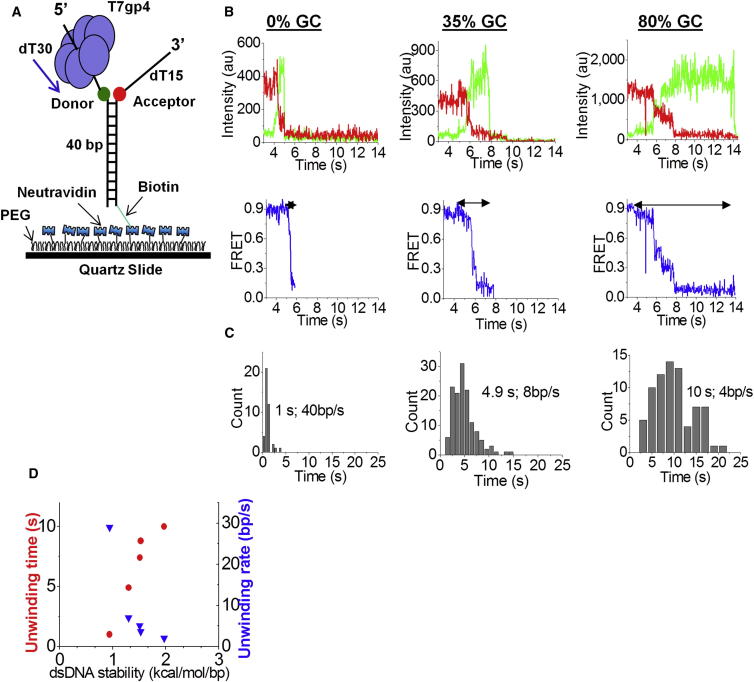
The Unwinding Rate of T7 Helicase Depends on Base Pair Stability (A) T7 gp4 was loaded on a 40 bp DNA with (dT)n tails containing donor (Cy3) and acceptor (Cy5) dyes, and bound to a PEG-coated surface via biotin-neutravidin interaction. (B) Cy3 and Cy5 intensity traces during unwinding for one molecule on mixed, 100% AT, and 80% GC sequences (top panel); calculated FRET efficiency versus time for the fluorescence intensity traces (bottom panel). (C) Dwell-time histograms during unwinding. The arrows on the FRET traces indicate the intervals at which the dwell times were measured; 50 molecules were used to build the histograms. The data are representative of multiple experiments. (D) Unwinding time (red circles) and rate (blue triangles) versus base pair stability. Five sequences were used to plot the graph (Table S1). See also Figure S1.

We probed the activity of T7 helicase using a single-molecule unwinding assay (Myong et al., 2007, Yodh et al., 2009) based on smFRET (Ha et al., 1996). We used forked substrates, where the Cy3 (donor) and Cy5 (acceptor) fluorophores were introduced at the ss/ds junction of the DNA with a 40 bp duplex (Figure 1A). The placement of fluorophores at the ss/ds junction does not alter the unwinding behavior (Pandey et al., 2009). The DNA was tethered to a polymer-treated quartz surface via biotin-neutravidin interaction (Figure 1A). After assembling T7 helicase on the DNA in the presence of 2 mM dTTP but no Mg(II), we initiated the unwinding reaction by flowing a solution containing 4 mM Mg(II) and 1 mM dTTP. This method of initiation served to remove the unbound protein in solution and enabled us to observe DNA unwinding catalyzed by prebound proteins only. Before unwinding starts, the donor and acceptor fluorophores are close together and therefore FRET is high (Figure 1B). As the unwinding reaction proceeds, the time-averaged distance between the fluorophores increases, resulting in a FRET decrease over time (Figure 1B; Ha et al., 2002, Myong et al., 2007, Yodh et al., 2009). When the donor-labeled strand departs from the surface after complete unwinding, the total fluorescence signal drops to the background level because the acceptor is not excited efficiently by the excitation laser at 532 nm.

To quantify the unwinding behavior, we measured the total unwinding time, which is the duration of the time interval from the moment FRET starts to decrease until the moment the total fluorescence signal disappears (Figure 1B, bottom panel, marked with arrows), and plotted the distributions (Figure 1C). A 10-fold difference in the unwinding time from 0% GC sequence to 80% GC sequence was observed (1 and 10 s, respectively; Figure 1C). After the unwinding reaction has progressed to a certain point, the remaining base pairs may separate spontaneously (Jeong et al., 2004). Therefore, we employed the following alternative method to estimate the absolute unwinding rate: First, we measured the time it takes to change FRET from 0.9 (the average value before unwinding starts) to 0.3. As we will show below, 0.3 FRET corresponds to ∼10 bp unwound (Figure S1). Dividing 10 bp by the average time it takes to unwind 10 bp gives the unwinding rate. We then plotted the total unwinding rate for five different substrates and plotted it against Δ*G*/bp (dsDNA stability) calculated using the nearest-neighbor approach (Breslauer et al., 1986) and the HyTher web-based program (SantaLucia, 1998). When the unwinding rate is measured in this way, the difference between 0% GC and 80% GC becomes 13-fold. The results confirm that the unwinding rate decreases as the base pair stability of the duplex increases (Donmez et al., 2007). Moreover, the results further validate our single-molecule assay because the unwinding rates obtained are in agreement with previously reported rates obtained from ensemble measurements (Donmez and Patel, 2008, Donmez et al., 2007).

### Calibration of the Number of Base Pairs Unwound to FRET Efficiency

**Figure 2:**
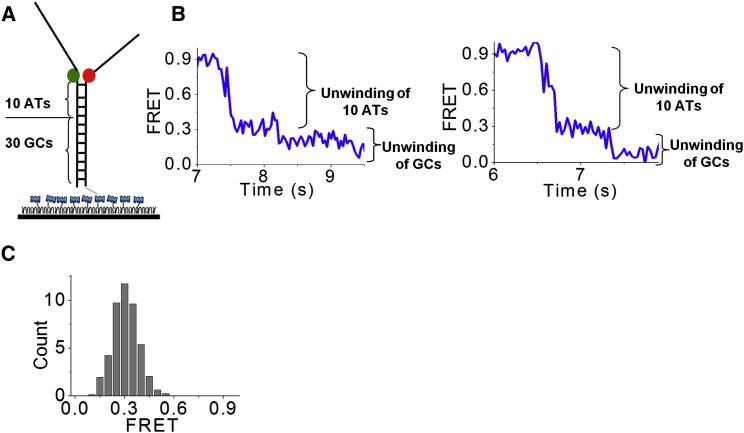
Calibration of the Number of Base Pairs Unwound to FRET (A) DNA construct schematics. (B) Representative calculated FRET efficiency versus time traces for 10 AT and 30 GC substrates (right and left panels). (C) FRET histogram. The arrows indicate the region of the FRET values used to build the histogram; >50 molecules were used to build the histogram. The histogram peaks at 0.3 FRET.

In order to make an approximate assignment of FRET values to the number of base pairs unwound, *N*_uw_, we performed the unwinding experiment using a substrate with ten contiguous adenine-thymine (AT) base pairs followed by 30 contiguous GC base pairs in the duplex region (Figure 2A; Table S1). After the unwinding reaction was initiated, FRET dropped rapidly to ∼0.3 in ∼0.9 s, likely due to unwinding of the AT base pair block, followed by a slow decrease to the lowest value (Figure 2B). To estimate the FRET value for *N*_uw_ = 10, we measured FRET values during the 1 s window after the initial, rapid FRET drop had ceased, and found that their histogram (>50 molecules) peaked at 0.3 (Figure 2C). Therefore, we will assume below that FRET is ∼0.3 for *N*_uw_ = 10.

### Unwinding Step Size of T7 Helicase

**Figure 3:**
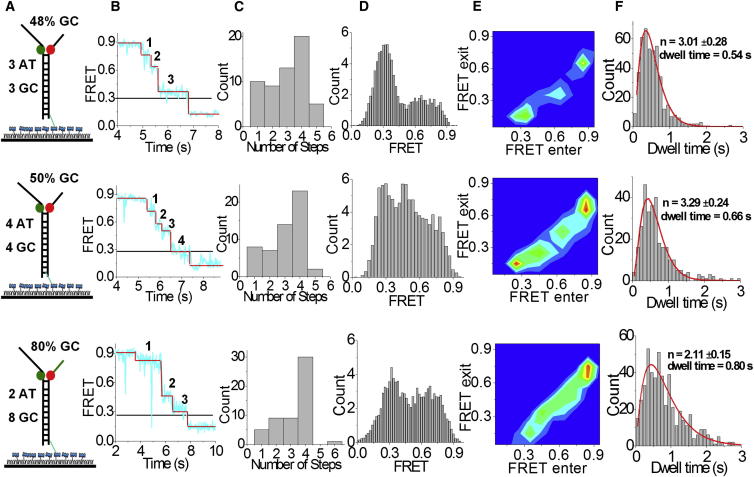
Analysis of Step Size and Stepping Kinetics (A) DNA sequences are made of repeats of n AT base pairs followed by n GC base pairs where n=3, 4, or 5. (B) A step-finding algorithm was used to measure FRET values and dwell times of the pauses for a single molecule. Three sequences with different GC content (from top to bottom: 48%, 50%, and 80%) were used for analysis. The step size was measured by counting the number of steps (n) until FRET reached a value of 0.3, and then dividing 10 by n. (C) Histogram of the number of steps that occurred for each substrate. (D) FRET histograms during unwinding; >50 molecules were used to build the histograms. (E) FRET values obtained from 80 molecules from each substrate were combined to make the transition density plot (TDP). (F) Gamma distribution fitting of the collected dwell times at each pause for >50 molecules.

Although all AT sequence DNA was unwound rapidly without any evidence of steps or pauses, DNA sequences containing ≥35% GC base pairs showed clear evidence of steps (Figure 1B). Because the apparent steps may be caused by GC base pairs that are slower to unwind (i.e., the steps may be sequence-dependent pauses rather than elementary steps), we analyzed in detail three substrates with a stretch (2, 3, or 4 bp) of AT base pairs followed by a stretch of GC base pairs in a repeating pattern with 48%, 50%, and 80% GC content, respectively (Figure 3A). We observed that T7 helicase unwound all three substrates in a stepwise manner, indicated by the plateaus observed during the FRET decrease (Figure 3B). For a minor fraction of molecules, FRET decreased with no detectable pause or only a single pause. However, in the majority of molecules, regardless of the sequence, three or four pauses could be visually identified, corresponding to four or five steps, respectively, in reaching from the highest to the lowest FRET values. We observed two or three pauses (or three or four steps) by the time FRET reached a value of ∼0.3, corresponding to *N*_uw_ = 10 (Figures 3B and 3C). Therefore, we can deduce that the pauses were separated by ∼2–3 bp. However, FRET histograms during the FRET decrease did not show distinct peaks, suggesting that there are not well-defined FRET states that are visited during unwinding, as would be expected if pauses occur when the helicase encounters a boundary caused by GC base pairs (Supplemental Experimental Procedures; Figure 3D).

To further quantify the pausing behavior, we used an unbiased step-finding algorithm that generated the average FRET value for each pause and its dwell time (Kerssemakers et al., 2006, Myong et al., 2007). We then built transition density plots (McKinney et al., 2006) that represented the two-dimensional histogram for pairs of FRET values, determined using the step-finding algorithm, before and after each pause (Figure 3B). The transition density plots also illustrate that the pauses during unwinding did not occur at well-defined FRET states (Figure 3E). Furthermore, the histogram of pause durations determined using the step-finding algorithm showed a nonexponential distribution with an initial lag phase, and the average pause duration increased with the increase in GC content (Figure 3F). We fit the data into a gamma distribution ((*Δt*)^*N*–1^ exp(–*kΔt*), where *Δt* = dwell time, *k* = rate of hidden stepping within a dwell, and N = number of hidden steps), and the fit gave N = 2 or 3, suggesting that each ∼2–3 bp step is composed of two or three hidden kinetic steps (Figure 3F). Hence, our data indicate that there are two or three hidden steps before the burst of unwinding ∼2–3 bp of DNA, suggesting an elementary unwinding step size of 1 nt.

### T7 Helicase Slows Down and Moves Backward during Unwinding at Lower dTTP Concentrations

**Figure 4:**
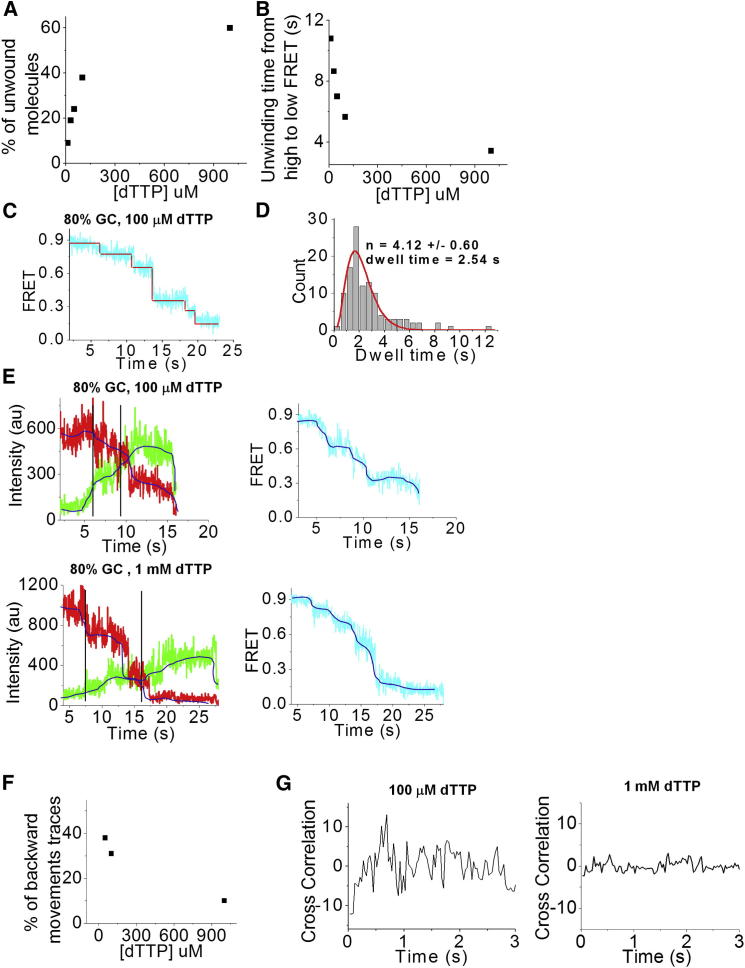
Dwell Time of Pauses with Lower dTTP Concentration and Backward Movements (A) Percentage of unwound molecules as a function of [dTTP]. (B) Unwinding time from high FRET to low FRET as a function of [dTTP]. (C) A step-finding algorithm was used to measure FRET values and dwell times of each pause for a single molecule. Experiments were performed using 100 μM dTTP on an 80% GC substrate. (D) Gamma distribution fitting of the collected dwell times at each pause for >50 molecules. (E) Cy3 and Cy5 intensity trace during unwinding on an 80% GC sequence, depicting backward movements by the T7 helicase during unwinding at 100 μM [dTTP] (top left panel), and Cy3 and Cy5 intensity trace displaying unwinding without backward movements at 1 mM [dTTP] (bottom left panel). The lines indicate the areas selected to calculate cross-correlation. Top and bottom right panels: calculated FRET efficiency versus time for the fluorescence intensity trace. Smoothed curves that were used for cross-correlation analysis are depicted in blue (see Supplemental Experimental Procedures). (F) Percentage of backward-movement traces as a function of [dTTP]. (G) Average cross-correlation curve from 50 molecules for 100 μM (left panel) and 1 mM dTTP (right panel). See also Figure S2.

To test whether the apparent step size of 2–3 bp persists even when unwinding becomes slower, we performed unwinding experiments on the 80% GC substrate with lower dTTP concentrations during T7 helicase assembly and unwinding (100 μM, 50 μM, 30 μM, and 10 μM). The number of molecules that showed unwinding dropped from ∼60% to ∼10% when [dTTP] was reduced from 1 mM to 10 μM, with the midpoint occurring at ∼75 μM (Figure 4A). This trend is consistent with the requirement for dTTP to assemble the ring helicase on the DNA. Among the molecules that showed unwinding, the average time it took for FRET to decrease from maximum to minimum values increased from 3.5 s to 11 s when [dTTP] was lowered from 1 mM to 10 μM (Figure 4B). Because of the much reduced yield of unwinding at 50 μM or lower dTTP, we focus on data obtained with 100 μM dTTP. We used the step-finding algorithm to measure the dwell time of each pause for molecules that showed a monotonic FRET decrease during unwinding, representing ∼70% of unwinding events (Figure 4C). The dwell histogram showed a nonexponential behavior and could be fit with gamma distribution, yielding N = 4.12 ± 0.6, similar to the 1 mM dTTP case (Figure 4D). The average dwell time was three times longer with 100 μM dTTP (2.5 s) than with 1 mM dTTP (0.8 s; Figures 3F and 4B). Therefore, the rate-limiting step required for an escape from a paused state likely involves dTTP binding.

Furthermore, ∼30% of the unwinding traces with 100 μM dTTP showed FRET fluctuations that appeared nonmonotonic, indicating partial reannealing of DNA and backward movements by the T7 helicase (Figures 4E and S2). Experiments performed using 50 μM dTTP showed a higher fraction (∼38%) of the traces with similar fluctuations in the FRET values, further demonstrating that backward movements are more prevalent at lower [dTTP] (Figure 4F). A cross-correlation analysis of donor and acceptor intensity time traces after subtracting the median filtered (2 s sliding window) intensities from the raw data (Supplemental Experimental Procedures) showed a decay starting from a negative value, confirming that the donor and acceptor’s intensity fluctuations are indeed anticorrelated (Figures 4E, top panel, 4G, and S2). In contrast, the cross-correlation curve calculated from the data obtained with 1 mM dTTP showed essentially zero amplitude without any negative values at early time points (Figures 4E, bottom panel, and 4G). From visual analysis of the 1 mM dTTP data, we found that <10% displayed a monotonic FRET decrease using unwinding (Figure 4E). Thus, backward movements occur at a higher frequency at lower dTTP concentrations.

## Discussion

Previous structural, ensemble, and single-molecule data (Johnson et al., 2007, Liao et al., 2005, Singleton et al., 2000) on T7 helicase suggested a sequential dTTP hydrolysis model akin to the staircase model proposed for E1 helicase (Enemark and Joshua-Tor, 2006) and Rho helicase (Thomsen and Berger, 2009), with the exception of a step size that is either larger than 1 bp (Johnson et al., 2007) or is variable depending on the GC content of DNA (Donmez and Patel, 2008). Using optical-tweezers analysis, Johnson et al. (2007) could not observe unwinding steps directly due to limited resolution (∼10 bp), and obtained a step size estimate of ∼2 bp based on mathematical modeling. In addition, a recent report on the crystal structure of DnaB helicase with ssDNA suggested a 2 bp unwinding step size per ATP hydrolyzed based on the occupancy of 2 nt per DNA-binding loop (Itsathitphaisarn et al., 2012). Our smFRET data provide direct evidence for a stepping behavior of hexameric helicase during unwinding. We obtained evidence of discrete unwinding steps of 2–3 bp, which is in apparent agreement with the 2 bp unwinding steps proposed for T7 helicase and DnaB. However, our kinetic analysis showed that our data are inconsistent with models that involve 2 bp steps per nucleotide hydrolysis because the lag phase in dwell-time histograms suggests two or three hidden steps per unwinding step. The data as a whole, therefore, are consistent with a coupling ratio of 1:1 between base pairs unwound and dTTP hydrolyzed, as demonstrated by Pandey and Patel (2014), assuming that the hidden steps correspond to dTTP hydrolysis events.

The unwinding of several base pairs in a burst with hidden kinetic steps is reminiscent of the spring-loaded mechanism of NS3 helicase (Myong et al., 2007), and elastic coupling between RNA degradation and unwinding of yeast exoribonuclease Rrp44 (Lee et al., 2012). The concept that nucleic acid motors can have a hierarchy of different-sized steps or can accumulate elastic energy before transitioning to a subsequent phase (Ali and Lohman, 1997, Bianco and Kowalczykowski, 2000, Kapanidis et al., 2006, Lucius et al., 2002, Moffitt et al., 2009, Myong et al., 2007, Revyakin et al., 2006, Schwartz et al., 2009) is further supported by our data. We also considered the possibility that the helicase unwinds 1 bp per step, but the unwound strand is released from the helicase only after two or three 1 bp steps. However, this mechanism is less likely because the unwinding speed is strongly dependent on the GC content. If the observed DNA unwinding is limited by asynchronous strand release, this step would not be dependent on the stability of dsDNA.

The 2–3 bp step size of unwinding is similar to the 3 bp and 4 bp unwinding step sizes estimated for DNA and RNA unwinding by NS3 and Rrp44, respectively. We do not know why such different classes of enzymes show nearly identical step sizes of ∼3 bp. Because 3 nt are also bound by RecA/Rad51 to form a recombination intermediate and are recognized as codons during translation, we speculate that the number 3 may come from intrinsic properties of the nucleic acids themselves.

In addition, we observed behavior consistent with backward movements at lower [dTTP] (Figure 4E), which is evidence of localized rezipping for hexameric helicases. Previous studies observed localized rezipping and reinitiation of unwinding on the scale of 5–20 bp for nonhexameric helicases using smFRET (Myong et al., 2007, Yodh et al., 2009) and, more recently, optical and magnetic tweezers (Klaue et al., 2013, Qi et al., 2013). The localized rezipping at low dTTP concentrations probably had a different origin compared with the large (200–1,000 bp) helicase backslips observed using ATP (Sun et al., 2011), because the large-scale backward movements were not observed with dTTP only.

**Figure 5:**
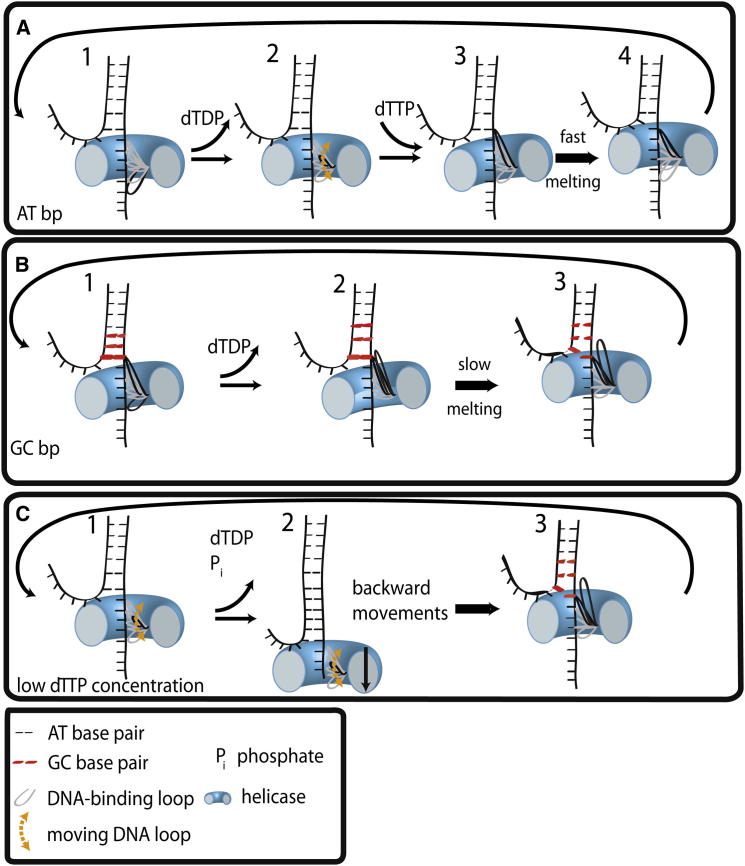
T7 Helicase Unwinding Model (A) dsDNA unwinding of AT base pairs. Only the DNA-binding loop of the rearmost position is shown, which is released upon dTDP release. Next, dTTP binds and the released loop binds the DNA at the foremost position. The fast melting of AT base pairs enables the enzyme to move forward. (B) dsDNA unwinding of GC base pairs. Slow melting of GC base pairs slows down the enzyme. When the enzyme encounters GC base pairs, reengagement of the disengaged loop with the DNA ahead is hindered due to steric mismatch between dsDNA and central helicase pore. Subsequent dTTP hydrolysis and product release by the next rearmost subunits build enough strain in the system to eventually cause 2–3 bp unwinding in a burst. (C) dsDNA unwinding at low dTTP concentrations. The DNA-binding loop does not readily bind to the DNA because the subunit remains in the dTTP unbound state. Subsequent dTTP hydrolysis and dTDP release at other subunits leads to the detachment of all of the DNA-binding loops and backward movements by the enzyme, and the DNA rezips. Once a new set of contacts is created by the enzyme on the tracking strand, the enzyme can move forward and unwind DNA.

Based on our data and the aforementioned ensemble and single-molecule studies and crystal structures, we propose the following unwinding model for T7 helicase, which may be applicable to other hexameric helicases. Six subunits are bound to ssDNA and dTTP in different hydrolysis states, with the rearmost subunit in the product state (Figure 5A). T7 helicase binds to DNA with high affinity in the presence of dTTP (Hingorani and Patel, 1993). Upon dTTP hydrolysis and product release by subunits around the ring, the rearmost subunit bound to the n^th^ nucleotide on the DNA loses contact with a backbone phosphate. In the normal catalytic cycle, the disengaged DNA-binding loop will assume the leading position and reengage with the (n+6)^th^ nucleotide coincidently with binding a new dTTP (Figure 5A, steps 2 and 3). If the subsequent base pairs to be unwound are AT base pairs, they will melt rapidly (Figure 5A, step 4), facilitating such reengagement. Then, the next subunit bound to the (n+1)^th^ nucleotide will detach from the DNA upon its own product release and rebind to the (n+7)^th^ nucleotide and so on, and such repeated cycles will cause unwinding of DNA in single base pair steps via coordinated sequential hydrolysis (Crampton et al., 2006, Enemark and Joshua-Tor, 2006, Liao et al., 2005, Singleton et al., 2000, Thomsen and Berger, 2009). However, when the enzyme encounters GC base pairs, reengagement of the disengaged loop with the DNA ahead is hindered due to steric mismatch between dsDNA and central helicase pore (Figure 5B, steps 1 and 2). Subsequent dTTP hydrolysis and product release by the next rearmost subunits will build enough strain in the system to eventually cause unwinding of several base pairs in a burst, resetting the system (Figure 5B, step 3). Our results indicate that this happens on an average after every 2–3 bp unwinding steps, but in a stochastic manner.

At subsaturating dTTP concentrations, it is difficult for the disengaged DNA-binding loop to reengage with the next backbone phosphate. Upon subsequent hydrolysis of dTTP and dTDP release in the other subunits, all of the DNA-binding loops may become detached from the tracking strand. As a result, the enzyme will move backward and the DNA will rezip (Figure 5C, steps 1 and 2). Once a new set of contacts is created by the enzyme on the tracking strand, the enzyme can move forward (Figure 5C, step 3).

We propose the above model based on our data from GC-rich sequences. The same unwinding mechanism may also apply to AT-rich sequences; however, a conclusive answer awaits measurements with higher spatiotemporal resolution.

## Experimental Procedures

### Protein, DNA Substrates, and Annealing

T7 helicase (gp4A′) protein was purified as described previously (Patel et al., 1992). Protein concentration was calculated by UV absorption in 8M guanidine hydrochloride using the extinction coefficients at 280 nm 0.0836 μM^−1^cm^−1^ for T7 gp4A′. DNA strands (Table S1) were purchased from Integrated DNA Technologies. Attachment of biotin at the 5′ end was done during DNA synthesis. Cy3 NHS-ester and Cy5 NHS-ester (GE Healthcare) were conjugated to an internal dT of single-stranded DNA strands via a C6 amino linker. DNA strands (5 μM final concentration) were annealed by heating complementary strands in T50 buffer (10 mM Tris, 50 mM NaCl, pH 8) to 90°C for 5 min and slowly cooling them for 2–3 hr to room temperature.

### Single-Molecule Unwinding Measurements of T7 Helicase

A quartz microscope slide (Finkenbeiner) and coverslip were coated with polyethyleneglycol (m-PEG-SVA-5000; Laysan Bio) to reject nonspecific binding of proteins (Ha et al., 2002), and biotinylated-PEG (biotin-PEG-SC-5000; Laysan Bio). A flow chamber for the single-molecule measurements was assembled as follows: After the coverslip and quartz slide were assembled (Roy et al., 2008), a syringe was attached to an outlet hole on the quartz slide through a tubing. All of the solution exchanges mentioned below were done by putting the solution (0.1 ml) in a pipette tip and affixing it in the inlet hole, followed by pulling of the syringe. The solutions were added in the following order: NeutrAvidin (0.2 mg/ml; Pierce) was applied to the surface and washed away with T50 buffer (10 mM Tris, 50 mM NaCl, pH 8). Biotinylated DNA (∼50–100 pM) in T50 buffer was added and washed away with imaging buffer (10 mM Tris, 50 mM NaCl, 0.1 mg/ml glucose oxidase [Sigma], 0.02 mg/ml catalase [EMDBiosciences], 0.8% dextrose, and 1% v/v 2-mercaptoethanol [Acros], pH 8). Next, T7 gp4 (50 nM hexamer) was loaded on the DNA with 2 mM dTTP and 5 mM EDTA in imaging buffer, and incubated for 10 min. After several seconds of imaging, the unwinding reaction was initiated by adding 1 mM dTTP and 4 mM free Mg(II) in imaging buffer. All measurements were done at room temperature (23°C ± 1°C).

## Supplemental Information

Document S1. Supplemental Experimental Procedures, Figures S1 and S2, and Table S1

Document S2. Article plus Supplemental Information
