# Supplementary material for: Single-Molecule Fluorescence Reveals the Unwinding Stepping Mechanism of Replicative Helicase
Source: Cell Rep. 2014 Mar 13;6(6):1037–45. doi: 10.1016/j.celrep.2014.02.022 (PMC3988844; doi:10.1016/j.celrep.2014.02.022)
Supplement: Document S1. Supplemental Experimental Procedures, Figures S1 and S2, and Table S1 [file mmc1.pdf]

Single Molecule Fluorescence Reveals the Unwinding Stepping Mechanism of Replicative Helicase

Salman Syed, Manjula Pandey, Smita S. Patel, and Taekjip Ha

Supplemental Experimental Procedures

Single Molecule Spectroscopy

Fluorescence signals arising from the unwinding reactions were imaged using a wide-field total internal reflection fluorescence microscope with 30-ms time resolution with an electron multiplying CCD camera (iXon DV 887-BI, Andor Technology). Cy3 on DNA was excited by an Nd:YAG laser (532 nm, 75 mW, Crysta-Laser) via total internal reflection. The fluorescence signals from donor and acceptor molecules were recorded using homemade software written in Visual C++. Single molecule traces were obtained from the recorded video file by using a script written in IDL (Research Systems, Boulder, CO). FRET values were calculated as the ratio between the acceptor intensity and the total (acceptor + donor) intensity after correcting for cross-talk between the donor and acceptor channels and subtracting the background using scripts written in Matlab (Roy et al., 2008).

Data Analysis

The total unwinding time was measured by visually inspecting the moment FRET starts to decrease until the moment total fluorescence signal disappears, and the time difference between the two points was designated as the total unwinding time. The unwinding time from FRET values of 0.9 to 0.3 was also measured by visually inspecting FRET decrease from 0.9 to 0.3, and the difference between the two points was designated as the unwinding time. FRET histograms during the period spanning the unwinding time were calculated and were normalized by the number of time points so that each molecule makes the same contribution to the final

FRET histogram. The stepwise decrease in FRET observed in unwinding and the dwell time of each step was processed via a stepfinder MATLAB program adopted from Kerssemakers et al (Kerssemakers et al., 2006; Myong et al., 2007) where the raw FRET traces are entered individually and the program finds steps. The transition density plots (McKinney et al., 2006) were derived from the data obtained from the stepfinder program and plotted in Origin (OriginLab). In order to characterize the backward movements of the enzyme during unwinding, we performed the cross correlation analysis after subtracting the gradually decreasing FRET signal as follows. The overall decrease in FRET during unwinding was approximated by applying a median filter with 2 seconds sliding window and was subtracted from the raw data. With this method, we were able to demonstrate that fluctuations faster than 2 seconds are anticorrelated if a low dTTP concentration is used.

Table S1: Sequences of the oligodeoxynucleotides, Related to Figures 1-4

Name	Sequences
**0% GC**	5'T_{30} Cy3 AATTATATTTAAATTTAAATATTAATTAATATATTAATAT<br>5' Biotin ATATTAATATATTAATTAATATTTAAATTTAAATATAATTCy5T_{15}
**35% GC**	5'T_{30}Cy3 GAGCGGATTACTATACTACATTAGAATTCAGAGTGTAGAG<br>5' Biotin CTCTACACTCTGAATTCTAATGTAGTATAGTAATCCGCTCCy5T_{15}
**48% GC**	5'T_{30} Cy3 ATAGGCATTGCGTATCCGTTAGCCAATGGCATTGCGTATG<br>5'Biotin CATACGCAATGCCATTGGCTAACGGATACGCAATGCCTATCy5T_{15}
**50% GC**	5'T_{30} Cy3 ATAAGGCCATTAGCGGTATTCCGGAATTGCCGTAATCGCG<br>5' Biotin CGCGATTACGGCAATTCCGGAATACCGCTAATGGCCTTATCy5T_{15}
**80% GC**	5'T_{30} Cy3 ATGCGCGCGCGTTCCGCGGCATGCCGGCGGTAGCGGCCGC<br>5' Biotin GCGGCCGCTACCGCCGGCATGCCGCGGAACGCGCGCGCATCy5T_{15}
**10 AT –<br>30 GC**	5'T_{30} Cy3 ATTATTATTTGCGGGGCGGGCGGGGCGGCGGGCGCGGGCG<br>5' Biotin CGCCCGCGCCCGCCGCCCCGCCCGCCCCGCAAATAATAATCy5T_{15}

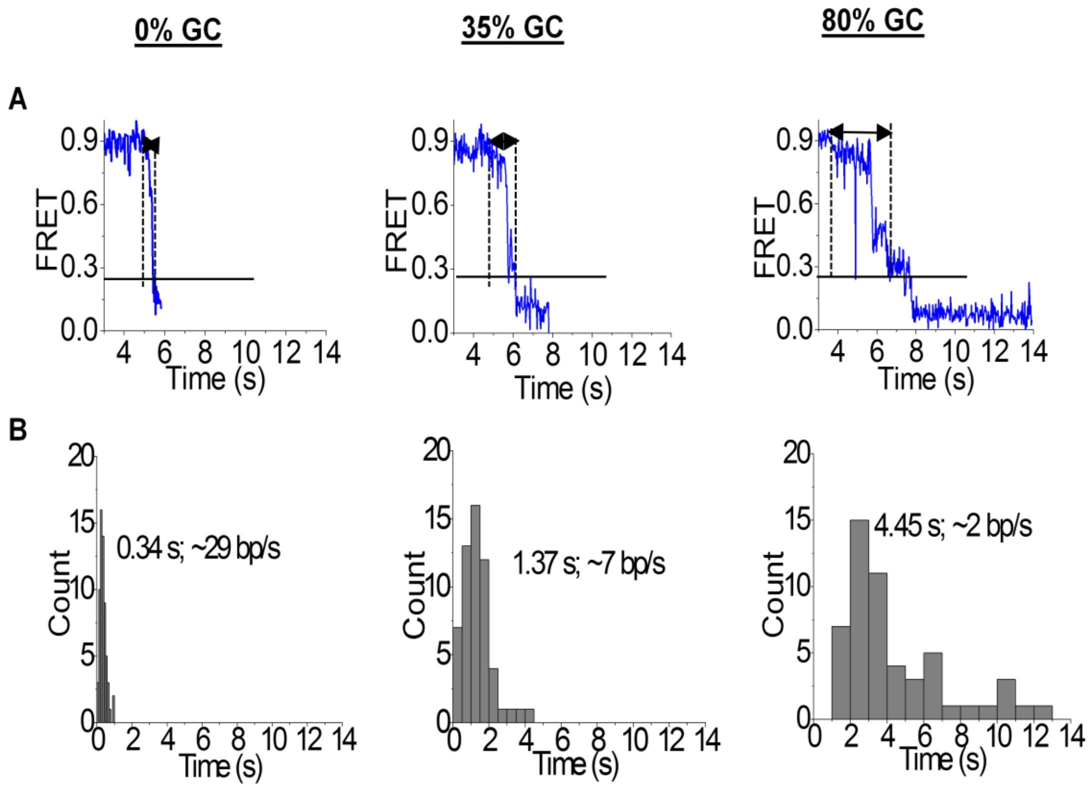

Figure S1, Related to Figure 1

(A) FRET time traces.
(B) Dwell time histograms during unwinding. The arrows on the FRET traces indicate the intervals at which the dwell times were measured; 50 molecules were used to build the histograms. The data is representative of multiple experiments.

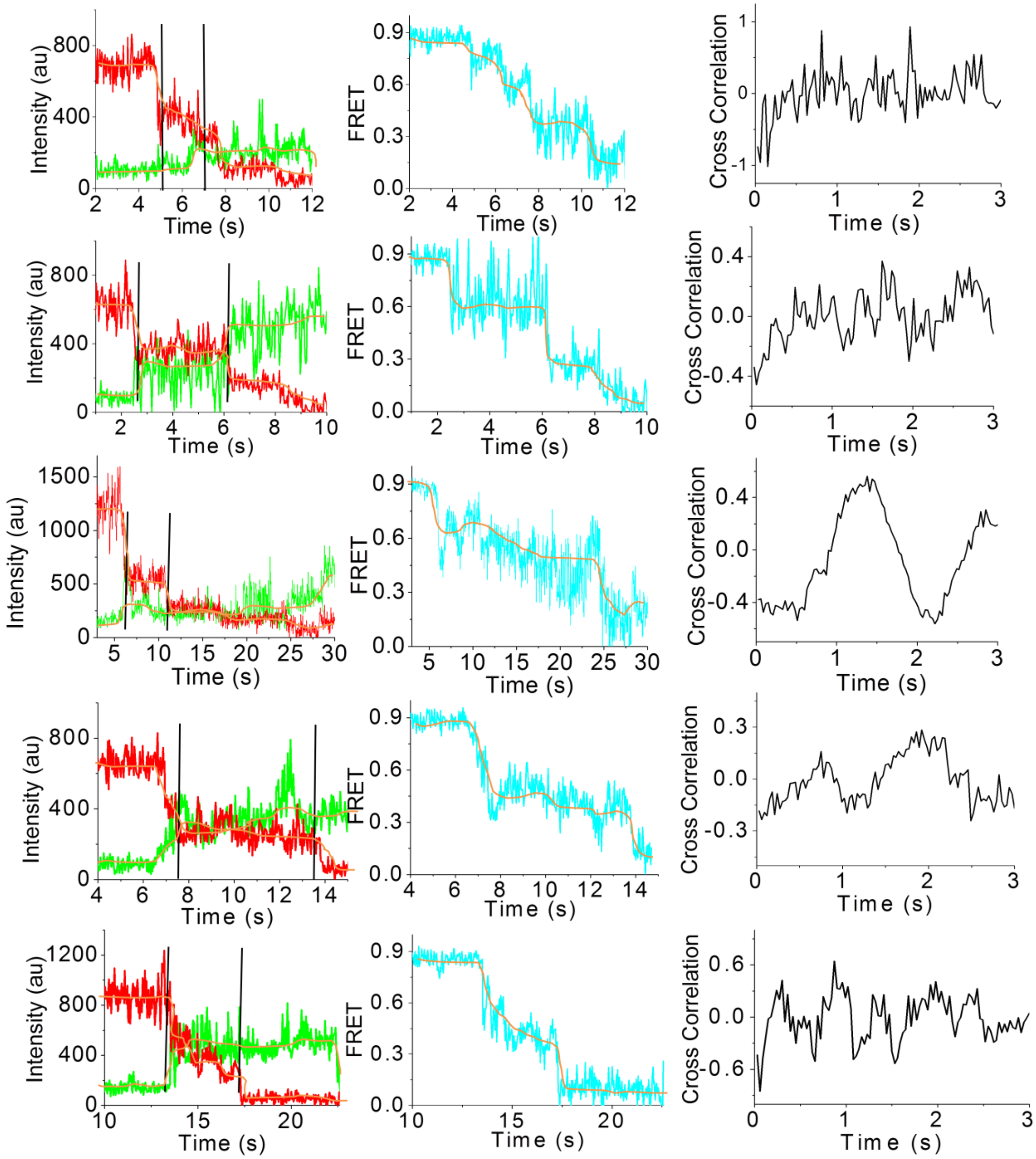

Figure S2, Related to Figure 4

More representative traces for backward movements for 100 μM dTTP (left and middle panel); cross correlation curves for individual traces; smoothed curves that were use for cross correlation analysis are depicted in blue (see Data Analysis).
